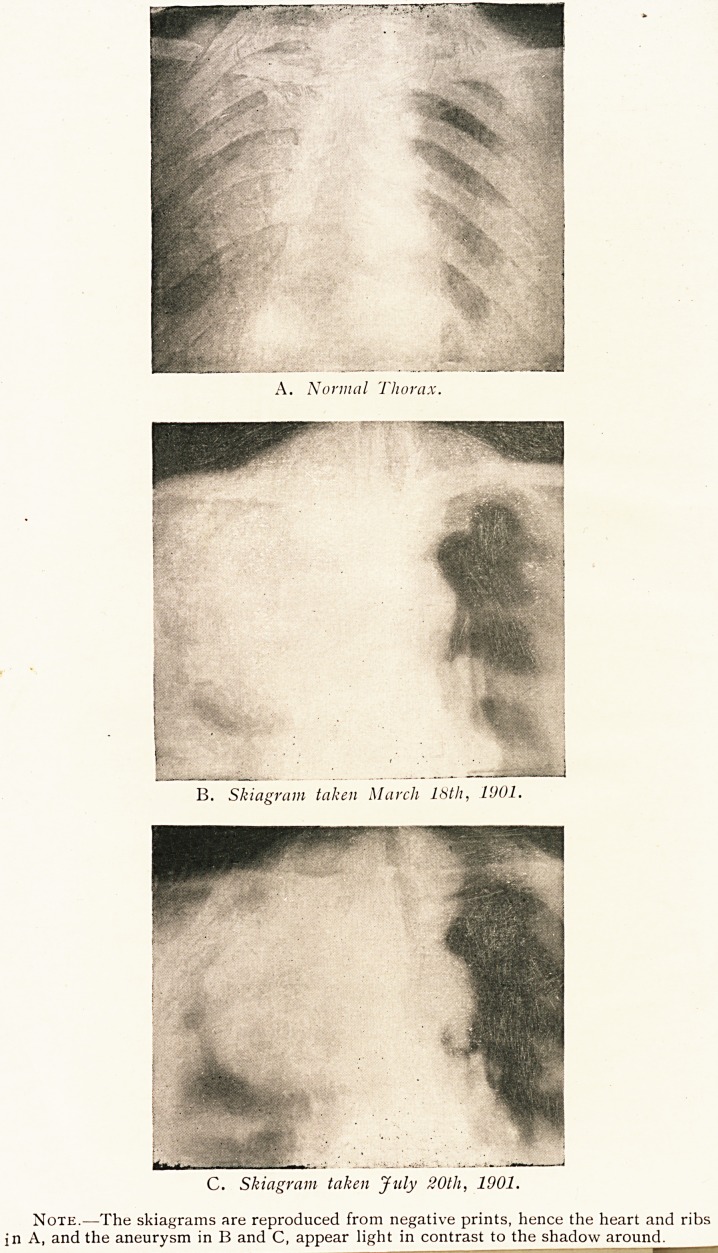# A Case of Paralysis of the Right Vocal Cord, Due to Thoracic Aneurysm

**Published:** 1901-09

**Authors:** Barclay J. Baron

**Affiliations:** Physician in Charge of the Throat and Nose Department of the Bristol General Hospital.


### A CASE OF PARALYSIS OF THE RIGHT VOCAL CORD, DUE TO THORACIC ANEURYSM.

BY

Barclay J. Baron, M.B. Edin.,

*Physician in Charge of the Throat and Nose Department of the Bristol General Hospital.*

Read before the Bristol Medico-Chirurgical Society, May 8th, 1901.

---

The patient, a man of 50 years of age, consulted me for hoarseness of voice in March of this year. As a matter of fact he was not hoarse, but his voice had that peculiar thin piping quality so often found where the movement of one or other cord is seriously interfered with.

He had suffered from this alteration of voice for a few months, but for about a year he had been feeling weak, was short of breath on exertion, was troubled with a certain amount of cough and expectoration, and he had lost several pounds in weight. On examining the larynx I found entire paralysis of the right vocal cord. His left pupil was distinctly dilated, and the left radial pulse was much smaller than the right one.

A. *Normal Thorax.*

B. *Skiagram taken March 18th, 1901.*

C. *Skiagram taken July 20th, 1901.*

NOTE.—The skiagrams are reproduced from negative prints, hence the heart and ribs in A, and the aneurysm in B and C, appear light in contrast to the shadow around.

Naturally this condition of things suggested an extensive aneurysm within the thorax. On stripping him and examining the chest, the only abnormality to be seen in front was a number of dilated subcutaneous veins on the right side of the sternum.

There was no bulging and no pulsation visible. On palpation no pulsation could be felt. On percussion resonance was only slightly impaired over the upper four ribs on the right side, nowhere amounting to dulness. On auscultation the breath sounds were practically unaltered over the whole of the front of the chest, and there was neither arterial nor cardiac abnormality audible. On examining the back at a point corresponding to the centre of the impaired resonance in front, tubular breathing could be heard over a limited area.

Dr. Markham Skerritt, who kindly saw the case with me, in consultation, confirmed all these observations.

Believing that skiagraphy would be of value, I asked Mr. James Taylor to take a skiagram. With the fluorescent screen we were able clearly to trace the course of a very large aneurysm, which, as the accompanying pictures show, involves the whole of the thoracic arch; it stretches out near to the axillary line on the right side and reaches practically to the clavicle, and bulges quite an inch to the left of the sternum.

The interest of the case centres in the fact that with almost no symptoms to distress the patient, and with almost no objective clinical signs, we yet have to do with an enormous aneurysm—an aneurysm of such a size as to seriously jeopardise the patient's life by rupture. The case also abundantly testifies to the extreme value of skiagraphy; in fact, I consider that where we have any considerable interference with the movement of vocal cords, especially if it be one cord only that is affected, unless it can be clearly determined that the cause of such crippling of the cord is of laryngeal or central origin, we ought always to avail ourselves of this aid to diagnosis. The treatment adopted during the past four months has been the usual one for such an intra-thoracic condition; viz., close confinement to bed, and lessening liquids to the lowest possible point that the patient could bear. Iodide of potassium was borne so extremely badly, that its administration had to be discontinued after a very short period. The vocal cord remains immovable; the left radial pulse is still distinctly smaller than the right one; the left pupil is, I think, somewhat less dilated, as compared with the right one, than it was four months ago. I am much indebted to Mr. James Taylor for kindly providing me with the accompanying excellent skiagrams.

## Figures and Tables

**A. B. C. f1:**